# A Multimodal Approach Combining Low-Intensity Shock Wave Therapy and Transcutaneous Electrical Nerve Stimulation for Refractory Chronic Pelvic Pain in Women

**DOI:** 10.7759/cureus.109939

**Published:** 2026-05-30

**Authors:** Salvador Rafael Solano-Sanchez, Elsa Patricia Maldonado-Miranda

**Affiliations:** 1 Obstetrics and Gynecology/Urogynecology, Hospital Ángeles Acoxpa, Ciudad de México, MEX; 2 Internal Medicine, Hospital Ángeles Acoxpa, Ciudad de México, MEX

**Keywords:** chronic pelvic pain, low intensity shock wave, pelvic floor rehabilitation, shock wave therapy, transcutaneous electrical nerve stimulation

## Abstract

Introduction

Chronic pelvic pain is a prevalent and multifactorial condition that significantly impacts quality of life. Multimodal management strategies targeting different pain pathways may offer improved clinical outcomes. This exploratory study aimed to evaluate whether a structured multimodal pelvic floor rehabilitation protocol combining low-intensity shock wave therapy (LiSWT) and transcutaneous electrical nerve stimulation (TENS) was associated with pain reduction in women with refractory chronic pelvic pain.

Methods

This study was designed as a single-arm pre-post study without a control group. It was conducted in 44 women with refractory chronic pelvic pain. All participants underwent a structured pelvic floor rehabilitation program consisting of 12 weekly sessions. TENS was applied weekly, and LiSWT was administered in eight sessions during the treatment period. Pain severity was measured with the Visual Analog Scale (VAS) before initiation of therapy and after completion of the rehabilitation program. Pre- and post-treatment VAS scores were compared using a paired-samples t-test.

Results

Final analysis included 44 women with refractory chronic pelvic pain. Mean pain scores decreased from 9.3 before treatment to 1.2 after treatment, demonstrating a statistically significant reduction.

Conclusion

The combination of LiSWT and TENS was associated with a clinically meaningful short-term reduction in pain among women with refractory chronic pelvic pain. These preliminary findings support the potential role of multimodal therapeutic strategies targeting different mechanisms involved in chronic pain. Further randomized controlled studies with long-term follow-up are required to confirm these results, isolate the contribution of each modality, and evaluate the durability of response.

## Introduction

Chronic pelvic pain refers to persistent or recurrent pelvic pain lasting more than six months and is frequently accompanied by hypersensitivity, urinary or bowel symptoms, and sexual dysfunction, in the absence of an identifiable organic cause [1]. This condition affects a substantial proportion of women worldwide, with an estimated prevalence of approximately 14%, and non-gynecologic etiologies may account for up to 80% of cases [2].

Chronic pelvic pain syndrome is considered a multifactorial condition involving potential contributions from urogynecologic, gastrointestinal, musculoskeletal, and neurological systems. Several mechanisms have been suggested as the pathophysiological basis of chronic pelvic pain: An infection process, a neurogenic inflammation, a disrupted blood flow to the pelvic area followed by decreased perfusion, and hyperactivity of the pelvic floor muscles. These mechanisms are unlikely to act independently and probably interact in the development and persistence of symptoms [3].

The management of chronic pelvic pain continues to represent a complex clinical problem and is associated with a considerable healthcare burden. Because symptoms frequently persist without a clearly identifiable cause, treatment is often challenging. In this context, multimodal strategies targeting different mechanisms involved in pain generation may offer greater clinical benefit than isolated interventions [4].

Beyond pain itself, this condition may substantially affect emotional well-being, social functioning, and overall quality of life [5]. Previous studies have reported anxiety in approximately 66% of affected women and depressive symptoms in nearly 63% [6].

Since chronic pelvic pain is a highly prevalent disease that significantly affects quality of life and current treatments offer limited effectiveness, we considered the possibility of applying another technology that could improve this problem. For this reason, low-intensity shockwave therapy was considered, which has been used in some urogynecological pathologies. Over recent decades, shock wave technology has progressively expanded into different medical fields and has been investigated as a non-invasive therapeutic option for several disorders.

Previous studies have explored the use of low-intensity shock wave therapy (LiSWT) in urogynecological and pelvic floor diseases, although the therapeutic efficacy and the molecular mechanism action is not clear. In fact, one of the first articles on these issues, although it is only a case report, was directly related to chronic pelvic pain [7].

Several mechanisms have been described in pelvic tissues: First, the use of low-intensity shock waves in rat models for the treatment of stress urinary incontinence resulted in improvement. This is associated with promoting angiogenesis and regeneration of the urethral sphincter [8]. Additionally, LiSWT has been shown to result in clinical improvement in overactive bladder after eight weeks of treatment. Although the therapeutic mechanism is not clear, it appears to be a promising treatment [9]. Furthermore, the use of low-intensity shock waves has been reported for the treatment of vestibulodynia. It is suggested that the treatment improves tissue mechanics and promotes the release of growth factors and anti-inflammatory factors. This could be related to the induction of angiogenesis and tissue regeneration [10]. Another study has also reported the effectiveness of this treatment for stress urinary incontinence. They also suggest that after eight weeks of treatment, urine leakage decreases, as well as urinary frequency, urgency, and nocturia [11]. According to this study, it seems that the time needed to observe pathophysiological changes in the pelvic floor is 8 weeks of treatment. Additional reports have suggested that LiSWT may improve symptoms associated with an overactive bladder. It has demonstrated clinical improvement in voiding volume as well as urgency, frequency, nocturia, and urinary incontinence [12].

With this information and background, considering that LiSWT could have a mechanism of action involved in pain pathways, we considered the possibility of complementing the management of chronic pelvic pain in women who were scheduled for pelvic floor rehabilitation with transcutaneous electrical nerve stimulation (TENS) (treatment that is reported as an alternative and that has efficacy around 70%) [13].

The justification for applying LiSWT to chronic pelvic pain is based on the fact that this treatment has been associated with mechanotransduction, neoangiogenesis, tissue hyperperfusion, inflammatory modulation, and tissue repair. These mechanisms may be relevant in the pathophysiology of chronic pelvic pain. However, their role in this specific population remains hypothetical, and confirmation will be required through other study designs that evaluate both biological and clinical outcomes.

Therefore, the primary objective of this exploratory study was to evaluate whether a structured multimodal pelvic floor rehabilitation protocol combining LiSWT and TENS was associated with a clinically relevant reduction in pain in women with refractory chronic pelvic pain. We hypothesized that this combined protocol would be associated with a significant reduction in pain scores after completion of the rehabilitation program.

## Materials and methods

A single-arm pre-post design without a control group was used in 44 women with chronic pelvic pain in a hospital setting. The study was conducted in a private urogynecology practice from January 2022 to December 2024, where patients were recruited using consecutive sampling. All evaluations were performed by trained clinicians following standardized procedures.

Women with refractory chronic pelvic pain who agreed to participate were considered eligible for inclusion. In this study, the term “refractory” was used to describe women who had persistent chronic pelvic pain despite previous conservative or medical treatments. A systematic and detailed characterization of the specific type, duration, and intensity of each previous treatment was not performed. Chronic pelvic pain was defined according to standardized international criteria.

Patients were excluded in the presence of neurological or psychiatric disorders, pregnancy, advanced pelvic organ prolapse (stage III or higher), or significant uterine/adnexal pathology; and elimination criteria included fewer than four LiSWT sessions and incomplete information.

The sample size was estimated using analytical methods based on the t-test to compare means of continuous variables within the same group before and after treatment. Specifically, the calculation was performed for a pre-post comparison of pain assessment using the Visual Analog Scale (VAS) before and after treatment, with a paired t-test. The aim was to detect an intragroup reduction greater than 60% in pain scores, corresponding to an estimated effect magnitude of 2.64 and a standardized effect size of 1.05. A one-sided alpha of 0.025 and beta of 0.20, equivalent to 80% statistical power, were used. Based on these assumptions, the minimum required sample size was 17 women. The final cohort included 44 women because all eligible patients treated during the study period who met the inclusion criteria and had complete pre- and post-treatment data were included in the analysis.

Participants completed a structured pelvic floor rehabilitation protocol composed of twelve weekly 45-minute sessions. TENS was applied once weekly for 30 minutes using a perineal patch with standard clinical parameters: 1 Hz and 200 µs. In addition, during this period, participants received eight LiSWT sessions. LiSWT was delivered once weekly during weeks 1 to 4, paused during weeks 5 and 6, and then resumed once weekly during weeks 7 to 10. LiSWT was administered using an abdominal approach, with the applicator placed in the suprapubic region. Each session delivered 1000 pulses, at an energy level of 0.09-0.1 mJ/mm² and a frequency of 160 pulses per minute, equivalent to 2.66 Hz, using the Omnispec LilyCare system (Medispec). Therefore, the remaining weeks of the 12-session program consisted of weekly TENS-based pelvic floor rehabilitation sessions without LiSWT.

The intervention was delivered following a structured clinical protocol with predefined parameters for session duration, stimulation frequency, pulse duration, energy level, number of pulses, and treatment site. Consistency of intervention delivery was pursued by applying the same therapeutic scheme to all participants. The equipment was used according to the manufacturer’s specifications and under standardized clinical parameters. Patients included in the final analysis were those who completed the treatment protocol and had complete pre- and post-treatment VAS data.

Urodynamic diagnoses were defined according to the terminology described in the joint report of the International Urogynecological Association (IUGA)/International Continence Society (ICS) on female pelvic floor dysfunction.

Pain severity was evaluated with VAS before initiation of therapy and after completion of the rehabilitation program. Continuous variables were expressed as mean ± standard deviation, whereas categorical variables were reported as frequencies and percentages. Pre- and post-treatment VAS scores were compared using a paired-samples t-test as the primary analysis. Statistical analyses were conducted using StatPlus Pro version 7.7.0, with statistical significance established at p < 0.05.

## Results

A total of 57 women with refractory chronic pelvic pain were initially enrolled. Five patients were excluded because of gynecologic pathology, neurological impairment, or psychiatric disease, and eight were removed from the final analysis because they completed fewer than four treatment sessions. In the end, 44 patients were analyzed. Changes in pain severity before and after therapy were evaluated using the VAS.

The demographic characteristics were as follows: age, 44.6 ± 14.3 years; symptom duration, 5.2 ± 6.8 years; and pregnancies, 1.2 ± 1.4. The most frequent urodynamic findings included bladder outlet obstruction/dysfunctional voiding (52%), detrusor overactivity (23%), normal urodynamic studies (20%), and urodynamic stress urinary incontinence (5%). Because bladder outlet obstruction/dysfunctional voiding was the most frequent finding in this cohort, the criteria used for this diagnosis were specified. Bladder outlet obstruction was characterized by a reduced urinary flow rate and/or the presence of an increased post-void residual volume, together with increased detrusor pressure. Dysfunctional voiding was defined as an intermittent and/or fluctuating flow rate due to involuntary intermittent contractions of the periurethral striated muscles or levator muscles during voiding in neurologically normal women.

**Figure 1:**
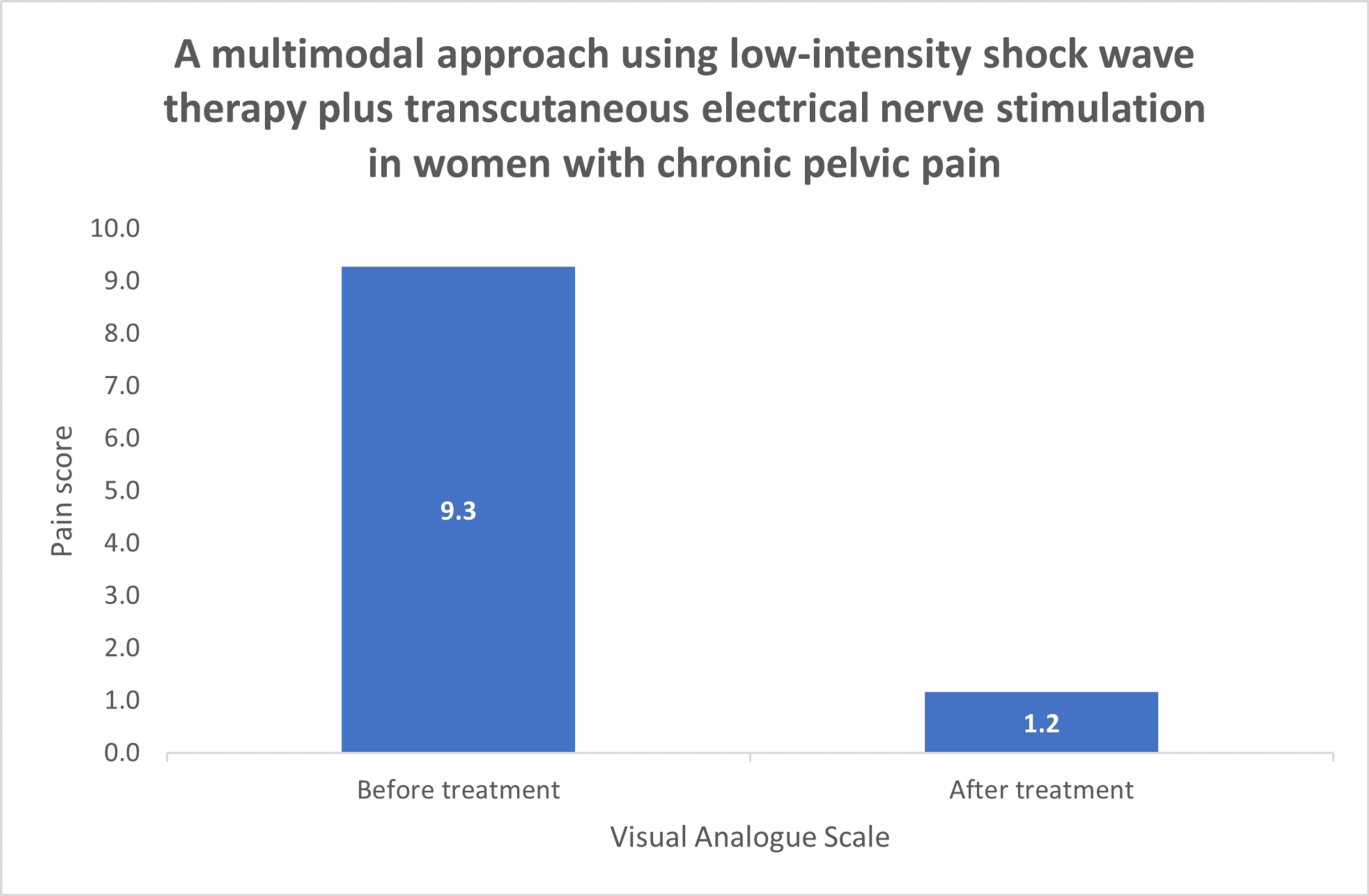
Pain scores before and after treatment (VAS) Pain scores in women with chronic pelvic pain were analyzed using VAS. Mean pain scores decreased from 9.3 before treatment to 1.2 after treatment, demonstrating a statistically significant reduction.

VAS scores obtained before and after treatment were compared using a paired-samples t-test. Normality of the paired differences was assessed by graphical inspection of the histogram prior to analysis. Mild asymmetry was observed in the distribution of paired differences; therefore, a Wilcoxon signed-rank test was also performed as a non-parametric sensitivity analysis. Mean pain scores decreased from 9.3 at baseline to 1.2 after treatment, demonstrating a statistically significant reduction in pain intensity (paired-samples t-test, t(43) = 35.57, p < 0.001). The mean difference in VAS scores was 8.11 points (95% CI: 7.65-8.57) (Table 1, Figure 1). The Wilcoxon signed-rank test confirmed the statistically significant reduction in VAS scores after treatment (Z = 5.78, p < 0.001).

**Table 1:** Pain scores before and after treatment (VAS) Paired t-test

VISUAL ANALOG SCALE (VAS)	N	Mean	SD	p value
Before treatment	44	9.3	0.9	< 0.001
After treatment	44	1.2	1.3	

## Discussion

To the best of our knowledge, no previous prospective study has evaluated the combination of LiSWT and TENS in women with chronic pelvic pain. Preliminary findings from this study were previously presented as a conference abstract [14]. In this exploratory single-arm study, the combined TENS-LiSWT protocol was associated with a marked and clinically relevant short-term reduction in pain. These findings suggest that this combined non-invasive approach may represent a promising therapeutic strategy for patients with long-standing symptoms who have frequently failed previous treatments. However, given the study design, these results should be interpreted as preliminary and hypothesis-generating.

The magnitude of pain reduction observed in this cohort suggests that addressing multiple pathophysiological mechanisms simultaneously may be clinically relevant in chronic pelvic pain. Rather than isolating the effect of each intervention, this study supports the concept of a multimodal therapeutic strategy in which TENS and LiSWT may act in a complementary manner. Chronic pelvic pain is widely recognized as a complex condition involving central sensitization, peripheral inflammation, and muscular dysfunction, and may therefore benefit from combined approaches targeting these different pathways. In addition, LiSWT has been associated with vasodilation, neoangiogenesis, improved tissue perfusion, anti-inflammatory effects, tissue remodeling, and reinnervation [15], which could contribute to pain improvement.

It is important to clarify that the multimodal rehabilitation protocol used in this study did not include stretching, down-training, or manual therapy. The intervention consisted specifically of TENS and LiSWT applied as part of a structured pelvic floor rehabilitation protocol. Therefore, the term “multimodal” in this study refers to the combination of these two therapeutic modalities within the same structured rehabilitation program.

Previous studies evaluating TENS alone in chronic pelvic pain have generally demonstrated partial or moderate improvement in pain symptoms. Current therapeutic options for chronic pelvic pain frequently provide incomplete symptom relief, supporting the need for multimodal strategies targeting different pathophysiological mechanisms. In this context, combining LiSWT with TENS may represent a rational treatment strategy. The marked reduction in pain observed in the present study supports the hypothesis that multimodal approaches targeting different pain pathways may provide additional clinical benefit [16-19].

Evidence regarding LiSWT in chronic pelvic pain remains limited, and most available studies have been conducted in male patients with chronic prostatitis/chronic pelvic pain syndrome (CP/CPPS). However, previous studies and meta-analyses have demonstrated that LiSWT may improve pain and quality-of-life outcomes in these patients, particularly when combined with other therapeutic modalities. These findings further support the concept that multimodal approaches targeting different pathophysiological mechanisms may provide additional clinical benefit in chronic pelvic pain [20-22].

Given the multifactorial pathophysiology of chronic pelvic pain, multimodal management seems to be one of the most promising approaches. It has even been reported that unimodal therapeutic options are mostly unsuccessful. In this context, the present study evaluated a structured multimodal protocol specifically combining LiSWT and TENS [4].

Although this is a single-arm pre-post study without a control group and larger controlled trials are needed to confirm these findings, the magnitude of improvement observed supports the clinical relevance of this therapeutic strategy. LiSWT appears to be a promising adjunct within the multimodal management of chronic pelvic pain.

One important limitation of this study is that both LiSWT and TENS were applied simultaneously within the same pelvic floor rehabilitation protocol, making it impossible to determine the individual contribution of each intervention. The observed improvement should therefore be interpreted as the clinical response associated with the combined LiSWT-TENS protocol, rather than as an isolated effect of either intervention.

The reduction in pain in this cohort was clinically relevant; however, it should be interpreted with caution. Given the single-arm, uncontrolled design, placebo effect, expectation bias, and regression to the mean cannot be ruled out. The findings should be interpreted as an association between the multimodal protocol and pain reduction, rather than as definitive evidence of causality. Selection bias may also be present because the cohort included women who agreed to participate and completed the structured multimodal protocol, which may limit the generalizability of the findings.

Additionally, the sample size was modest, and pain assessment was only performed at the end of the rehabilitation program. Therefore, no conclusions can be drawn regarding the medium- or long-term durability of symptom improvement.

This study should be interpreted within the context of its design. As a single-arm pre-post study without a control group, it does not allow determination of causality or the individual effect of each therapy. However, the results provide clinically relevant insight into the potential benefits of combining therapeutic modalities in real-world settings, where multimodal treatment is often necessary. In this context, LiSWT is particularly relevant because it may offer a biologically plausible, non-invasive way to target vascular, inflammatory, and tissue-repair mechanisms that are not directly addressed by conventional neuromodulatory approaches. Despite these limitations, this study provides preliminary clinical data on a combined non-invasive therapeutic approach in women with refractory chronic pelvic pain.

## Conclusions

In this exploratory single-arm study, a structured protocol combining LiSWT and TENS was associated with a clinically relevant short-term reduction in pain among women with refractory chronic pelvic pain. These preliminary findings suggest that this novel combination of non-invasive therapeutic modalities may represent a promising approach for this condition. Within this strategy, LiSWT is particularly relevant as an emerging non-invasive therapy with biologically plausible effects on vascular, inflammatory, and tissue-repair mechanisms. However, due to the absence of a control group, the inability to isolate the independent effects of each modality, and the lack of long-term follow-up, causality or durability of the response cannot be established. Future randomized controlled trials are required to confirm these findings, determine the specific contribution of each intervention, and assess long-term outcomes.
